# Reference value on daily living walking parameters among Japanese adults

**DOI:** 10.1111/ggi.13931

**Published:** 2020-05-06

**Authors:** Shuichi P Obuchi, Hisashi Kawai, Kenji Murakawa

**Affiliations:** ^1^ Tokyo Metropolitan Institute of Gerontology Tokyo Japan; ^2^ Taiyo Life Insurance Company Tokyo Japan

**Keywords:** daily living, reference data, walking speed

## Abstract

**Aim:**

Walking speed is closely related to numerous health outcomes. It has typically been measured in laboratory settings, where individuals can intentionally change their walking speed. It can be accurately measured in daily life using a smartphone global positioning system. We aimed to present a reference value on walking speed in daily life by sex and age.

**Methods:**

The data were obtained using a walking monitoring service involving global positioning system technology. A secondary data analysis was carried out. Four daily living walking parameters – daily living walking speed, daily living walking cycle, daily living step length and daily living cadence – of 8429 Japanese persons were measured in their daily life using a smartphone application.

**Results:**

The means (standard deviations) of daily living walking speed, daily living walking cycle, daily living step length and daily living cadence were 1.30 m/s (0.10 m/s), 1.02 s (0.06 s), 65.55 cm (5.38 cm) and 118.86 steps/min (6.76 steps/min), respectively. Notably, daily living walking speed in those aged >65 years was significantly slower than in those aged <65 years.

**Conclusions:**

The present reference values and age differences of daily living walking parameters can be used to compare daily living walking speed data measured by other devices, such as accelerometers. This could allow for a consensus on the definition of daily walking speed that can be utilized for assessing health outcomes among older individuals. **Geriatr Gerontol Int 2020; 20: 664–669**.

## Introduction

Walking speed is closely related to numerous health outcomes. For instance, it is a key criterion of frailty and sarcopenia among older persons.^1^, ^2^ In younger people, a decline in preferred walking speed is related to higher glycemic profiles among individuals with type 2 diabetes mellitus,^3^ and lower walking speed is a risk factor for cardiovascular events, cancer and all‐cause mortality, according to several systematic reviews.^4^, ^5^, ^6^, ^7^

However, walking speed is often measured under supervised conditions; for example, the time taken to walk a fixed distance (i.e. 2.5 m, 5 m or 10 m^8^, ^9^) at a comfortable pace or with maximal effort.^10^ When researchers require more precise measurements, they might use a motion capture system and force plates.^11^ The 6‐min walking distance is another type of measurement that is believed to better represent normal walking than other laboratory measurements, but its difference from walking during daily life has not been formally studied.^12^ Participants might walk faster when they know they are being observed than during daily life.

Walking speed is influenced by several factors. Finley *et al*. reported that walking speed varies according to site; that is, a shopping center, small commercial area, residential area or business area.^13^ Although 6‐min walking test results do not appear to differ between indoor and outdoor areas,^14^ walking speed has been found to be influenced by environmental noise,^15^ temperature,^16^ landscape and psychological factors in outdoor environments.^17^ Indeed, several studies suggest that walking characteristics measured in daily life using an accelerometer differ from those measured in a laboratory.^18^, ^19^, ^20^

Technological advancements, particularly smartphone‐based sensors and information communication technology, have enabled researchers to measure walking speed under natural conditions.^21^, ^22^ In previous research, we examined the test–retest reliability of a patented technology (patent number: WO2016043081) that measures walking speed in daily life using a smartphone global positioning system (GPS).^22^ Therein, we also compared this technology with measurement using a stopwatch (the gold standard), simultaneously carrying out 50 trials on a 50 m walkway at various speeds (1.08–2.10 m/s) and showing excellent validity (interclass correlation 0.945). These results enabled us to measure walking speed in daily life (DWS).

However, there is no consensus on the definition of DWS. Various definitions have been used in previous studies, such as the average walking speed during steady‐state periods of daily‐life gait,^23^ the average walking speed over several days^24^ and the daily life gait speed distribution itself.^25^ Furthermore, various devices can measure DWS. Accelerometers measure DWS using acceleration data and additional information through kinematic data or statistical estimation.^23^, ^24^, ^25^ Conversely, although GPS only measures DWS in outdoor settings, it accurately measures one’s position, rather than determining walking speed through an estimation. Although each method can remotely measure DWS, reference data do not exist. Such data are essential for developing a consensus on the definition of daily walking speed for assessing health outcomes in older individuals.

Therefore, we aimed to present reference data on DWS, by sex and age, using secondary data from large‐scale samples collected by a life insurance company that provides a GPS walking monitoring service to customers. Additionally, basic walking parameters, such as walking cycle (DCY), step length (DSL) and cadence (DCA), have been suggested as being associated with age.^26^ Thus, we also aimed to present reference values of DCY, DSL and DCA in daily life.

## Methods

### *Participants*

The study participants were life insurance plan subscribers. This study’s authors received anonymous data for all uses of the daily living walking measurement application provided by the company from 1 October 2016 to 31 March 2018. In other words, this study was carried out by analyzing substantial data collected through the walking monitoring service. The participants were 8429 Japanese individuals (2547 men, 5882 women) whose walking speeds during daily activities were measured across ≥50 walking bouts. Their mean (SD) age was 51.3 years (14.9 years), and they resided in all prefectures of Japan (Table S1). As the service involved monitoring walking speed in daily life, we did not receive participants’ detailed data, such as the presence or absence of disease. Therefore, the exclusion criteria were limited. The application is maintained and operated by InfoDeliver (Tokyo, Japan). Participants downloaded the application to their smartphones and used it of their own volition. All participants acknowledged that their data would be collected by the insurance company and used for the current study when they downloaded the application, and they agreed to participate in the study by clicking the approval button.

We explained the purpose of this study to the company, which then provided the anonymous data (i.e. walking parameters, prefecture, sex, age). The data provision to our institution was announced on the company’s website and in a company press release; all participants were provided the opportunity to opt out. This study was carried out in accordance with the Declaration of Helsinki and approved by the institutional review board and ethics committee of the Tokyo Metropolitan Geriatric Hospital and Institute of Gerontology (Acceptance No. K128, 2018).

### *Measurements*

The application used in this study employed a system that we previously evaluated for reliability and validity regarding daily living walking speed measurement, as described in the Introduction.^22^

The application, available on Android and iOS devices, measured participants’ walking trajectory using position information acquired from the smartphone GPS. When a stable straight walking trajectory was detected for >20 m, the walking speed was continually measured until it was interrupted. Once the application started, walking speed was measured in the background while the participant walked outdoors (as GPS is limited to outdoor use); the measurements were carried out without the participant’s awareness. This application also measured participants’ walking cycle and step length using a step counter. The step counter algorithm is kept private by the developer of each smartphone, but it generally identifies a walking event using the smartphone’s built in triaxial accelerometer and the number of steps counts during the walking distance. Step length is derived by dividing the walking distance by the number of steps; it is not calculated from the measurement of each step, but is the average step length in the walking distance. Similarly, walking cycle is the reciprocal cadence derived by dividing the average walking speed within the walking distance by the average step length. When participants had ≥50 measurements for a given parameter, the average value of those measurements was defined as the walking parameter in daily life. We calculated DWS, DCY, DSL and DCA. As the participants did not receive any specific instructions when using the application, the location of the smartphone (i.e. body, hands or bag) depended on each individual. Although this might have caused some error, we addressed it by using the average value of multiple measurements. As described in our previous study, because walking speeds that were measured ≥50 times for an individual were approximately normally distributed, the average value of those walking speeds can be regarded as the representative walking speed of an individual’s daily life.^22^ Thus, participants whose walking speeds were measured <50 times during the test session were excluded.

All measured walking parameters were transferred periodically to a designated server and routinely saved. Although we received user data from an application provided by a specific service, free applications using the same algorithm are available (Android: https://play.google.com/store/apps/details?id=com.infodeliver.android.hokoapp; iOS: https://itunes.apple.com/JP/app/id1331341017?mt=8).

### *Statistical analysis*

To statistically evaluate the walking speed distribution measured multiple times in daily life, we assessed the kurtosis and skewness of this distribution among all study participants. Mean kurtosis and skewness values close to 0 indicated a normal distribution. Kurtosis and skewness outliers were judged by the anomaly index of spss Statistics 25.0 J (IBM Japan, Tokyo, Japan), and one participant whose anomaly index was >50 was excluded in advance. For visual confirmation of normal distribution, 12 participants were randomly extracted, and histograms of walking speeds measured in daily life are provided as supplementary data. After the normality assessment, we calculated the means and SD for all walking parameters, including by sex and age group, which were investigated by a two‐way analysis of variance. All statistical analyses were carried out using spss Statistics 25.0 J.

## Results

The histograms of the 12 randomly selected participants’ walking speeds showed an essentially unimodal distribution (Fig. S1). The means of skewness and kurtosis for the walking speed distributions were 0.414 (SD 0.421) and 0.411 (SD 0.939), respectively (Fig. S2). The means (SD) of the walking parameters of all participants are shown in Table 1. Tables 2 and 3 show the means and SD for the walking parameters according to sex and age group. Among men, DWS was the highest (at 1.38 m/s) for those aged 25–29, 35–39 and 50–54 years, but the lowest (at 1.23 m/s) for those aged 80–84 years. Among women, DWS was the highest (at 1.29 m/s) for those aged <20 years and the lowest (at 1.20 m/s) for those aged 80–84 years.

**Table 1:** Descriptive statistics of walking parameters in daily life for all participants

Walking parameters for daily living	Minimum	Maximum	Mean	SD
DWS(m/s)	0.98	1.83	1.30	0.10
DCY (s)	0.83	1.25	1.02	0.06
DSL (cm)	47.64	92.46	65.55	5.38
DCA (steps/min)	96.93	145.15	118.86	6.76

Total (*n* = 8429). DCA, daily living cadence; DCY, daily living walking cycle; DSL, daily living step length; DWS, daily living walking speed; SD, standard deviation.

**Table 2:** Means and standard deviations of walking parameters in daily life by age among men

Men																		
Age group (years)	<20		20–24		25–29		30–34		35–39		40–44		45–49		50–54		55–59	
*n*	65		92		170		220		250		259		252		277		272	
	Mean	SD	Mean	SD	Mean	SD	Mean	SD	Mean	SD	Mean	SD	Mean	SD	Mean	SD	Mean	SD
DWS (m/s)	1.33	0.10	1.35	0.11	1.38	0.10	1.37	0.10	1.38	0.09	1.37	0.10	1.36	0.10	1.38	0.11	1.37	0.12
DCY (s)	1.03	0.05	1.05	0.05	1.05	0.05	1.05	0.06	1.05	0.05	1.04	0.05	1.04	0.06	1.03	0.05	1.03	0.06
DSL (cm)	68.29	4.95	70.82	4.88	71.97	5.03	71.28	4.79	71.59	4.72	70.84	4.75	70.50	4.92	70.76	5.67	69.97	5.44
DCA (steps/min)	116.86	5.67	114.74	5.95	114.99	5.78	115.07	6.26	115.34	5.86	115.92	6.08	115.96	6.33	116.84	6.16	117.68	6.71
Age group (years)	60–64		65–69		70–74		75–79		≥80									
*n*	209		203		151		83		44									
	Mean	SD	Mean	SD	Mean	SD	Mean	SD	Mean	SD								
DWS (m/s)	1.37	0.11	1.33	0.11	1.31	0.12	1.30	0.11	1.23	0.09								
DCY (s)	1.02	0.06	1.03	0.06	1.04	0.06	1.04	0.06	1.04	0.07								
DSL (cm)	69.38	5.17	68.02	5.48	67.46	5.42	67.16	5.03	63.78	5.43								
DCA (steps/min)	118.36	6.82	117.28	6.72	116.73	7.21	116.01	6.04	116.47	7.63								

Total *n* = 2547. DCA, daily living cadence; DCY, daily living walking cycle; DSL, daily living step length; DWS, daily living walking speed; SD, standard deviation.

**Table 3:** Means and standard deviations of walking parameters in daily life by age among women

Women																		
Age group (years)	<20		20–24		25–29		30–34		35–39		40–44		45–49		50–54		55–59	
*n*	48		110		256		353		410		501		682		722		830	
	Mean	SD	Mean	SD	Mean	SD	Mean	SD	Mean	SD	Mean	SD	Mean	SD	Mean	SD	Mean	SD
DWS (m/s)	1.29	0.07	1.27	0.08	1.26	0.09	1.26	0.08	1.26	0.09	1.28	0.09	1.27	0.09	1.28	0.09	1.28	0.09
DCY (s)	1.02	0.05	1.02	0.06	1.03	0.05	1.02	0.06	1.03	0.05	1.02	0.05	1.01	0.05	1.00	0.06	1.00	0.05
DSL (cm)	65.28	3.12	64.51	4.17	64.21	3.92	63.75	3.82	64.46	3.90	64.41	3.71	63.94	3.98	63.79	4.21	63.72	4.00
DCA (steps/min)	118.57	5.45	118.33	6.90	117.64	6.18	118.50	6.57	117.10	5.99	119.10	6.16	119.75	6.36	121.04	6.72	121.06	6.55
Age group (years)	60–64		65–69		70–74		75–79		≥80									
n	693		616		410		178		73									
	Mean	SD	Mean	SD	Mean	SD	Mean	SD	Mean	SD								
DWS (m/s)	1.28	0.09	1.26	0.09	1.25	0.09	1.23	0.09	1.20	0.09								
DCY (s)	1.00	0.05	1.00	0.06	1.00	0.06	1.01	0.06	1.03	0.05								
DSL (cm)	63.63	3.96	62.88	4.00	61.91	4.26	61.93	4.17	61.71	4.15								
DCA (steps/min)	120.86	6.24	120.44	6.73	121.60	7.03	119.32	6.55	116.90	5.97								

Total (*n* = 5882). DCA, daily living cadence; DCY, daily living walking cycle; DSL, daily living step length; DWS, daily living walking speed; SD, standard deviation.

**Figure 1:**
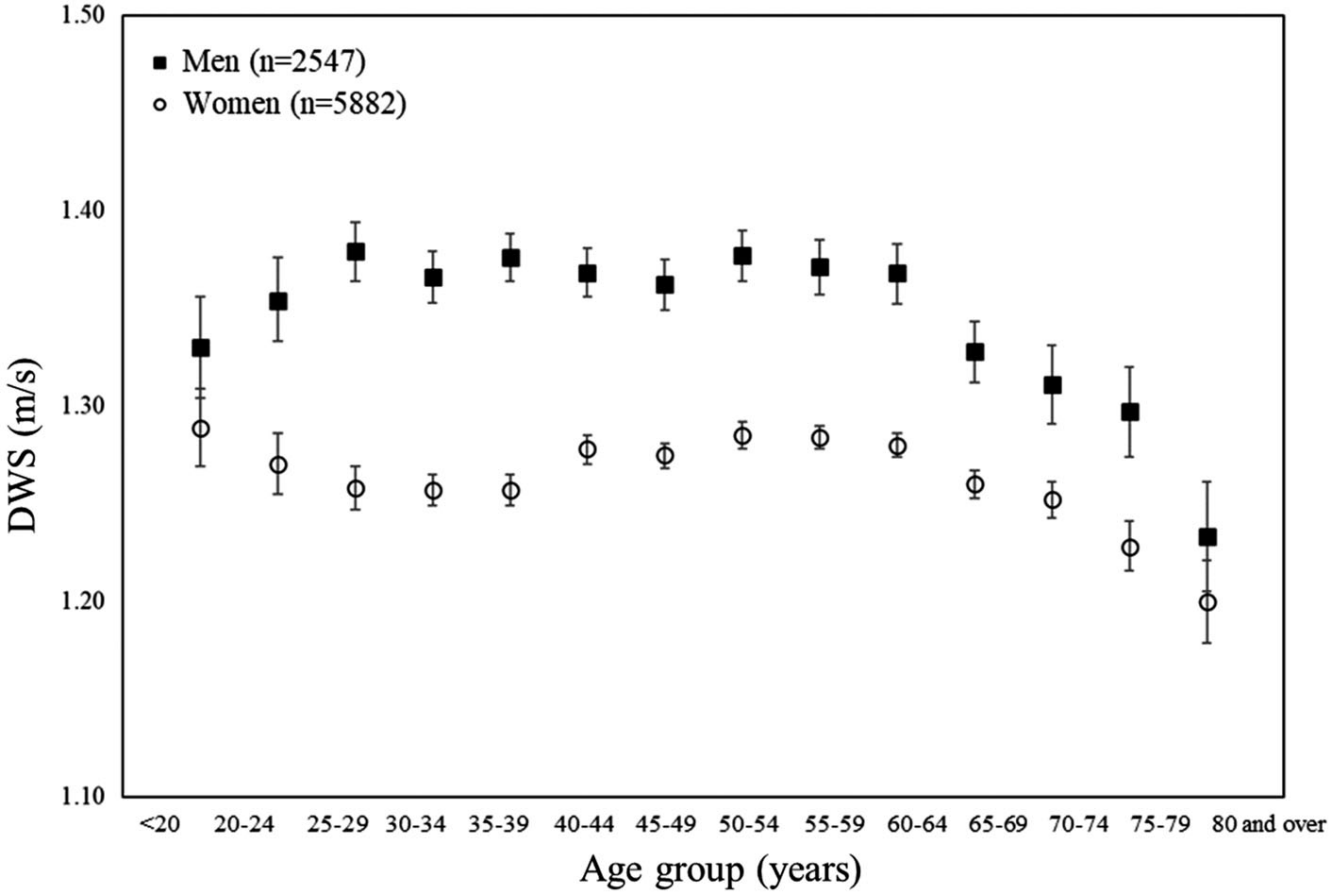
Average of daily living walking speed (DWS) according to sex and age groups (error bar: 95% confidence interval).

A two‐way analysis of variance of DWS, DCY, DSL and DCA according to sex and age groups recognized a main effect of sex (*F* = 851.15, 227.05, 1974.72 and 239.88, respectively, all p < 0.001) and a main effect of age group (*F* = 25.19, 18.50, 35.86 and 17.97, respectively, all p < 0.001) for all parameters. DWS showed significantly greater differences in those aged ≥65 years than in the younger groups among all participants (Figs 1, S3).

## Discussion

The present study provides reference data for DWS among Japanese individuals using a smartphone application relying on GPS. As all participants were recruited through a life insurance company and were limited to smartphone users, we examined their bias from the subscription rate of insurance service and smartphone ownership rates in Japan. Although the insurance subscription rate was 60.2% among those aged 20–29 years, and 52.4% among those aged ≥70 years, it was >75% in other middle‐aged groups.^27^ More than 70% of those aged <60 use smartphones, but just 44.6% of those aged 60–69 years, 18.8% of those aged 70–79 years and 6.1% of those aged ≥80 years do so.^28^ Thus, data from those aged >70 might be more biased; however, the present data came from participants from all Japanese prefectures, and are thus meaningful as a reference value of the daily living walking parameters.

DWS is affected not only by the environment, but also by walking duration, walking distance and distractions. As the present study used secondary data from smartphone applications, many factors were beyond our control. However, DWS was represented as the average value of walking speeds measured multiple times under various daily living situations, and our previous study supported this method’s test–retest reliability and validity. The present study statistically examined whether walking speed measured multiple times has a normal distribution for additional verification. The mean skewness and kurtosis values of all participants’ walking speed distribution were near zero, with the right tail being slightly longer and the center having many measurements (i.e. the distribution around the center was slightly pointed); these values largely indicated normal distributions. Thus, the results suggest that an average of walking speeds in daily life can be used as a representative daily life walking speed.

We also measured age and sex differences in each parameter. Men had a higher DWS than women (men 1.36 m/s, women 1.27 m/s), whereas women had a slightly lower DCY and DSL (DCY 1.04 s for men and 1.01 s for women; DSL 70.04 cm for men and 63.61 cm for women), but a higher DCA (men 116.37 steps/min, women 119.93 steps/min). The shorter DSL reflects that women are generally shorter than men, as shown in cohort studies of older Japanese individuals. Sex differences in walking parameters tended to be similar to those from a laboratory in a previous study that examined individuals aged 10–79 years.^26^

Compared with another previous laboratory study measuring the walking speed of a similar age group, DWS was lower than the laboratory walking speed (WSL) among individuals aged 65–69 and 70–74 years for all participants (men 65–69 years 1.39 m/s for WSL, 1.33 m/s for DWS; women 65–69 years 1.39 m/s for WSL, 1.26 m/s for DWS; men 70–74 years 1.33 m/s for WSL, 1.31 m/s for DWS; women 70–74 years 1.31 m/s for WSL; 1.25 m/s for DWS).^29^ Among individuals ≥75 years, DWS was higher than WSL in all participants. This suggests that the present study may might been biased by including particularly active older individuals. A recent study reported that DWS measured using an accelerometer was significantly lower than WSL.^23^ Whether DWS is actually higher than WSL should be explored in future work that assesses these parameters with the same participants.

**Figure 2:**
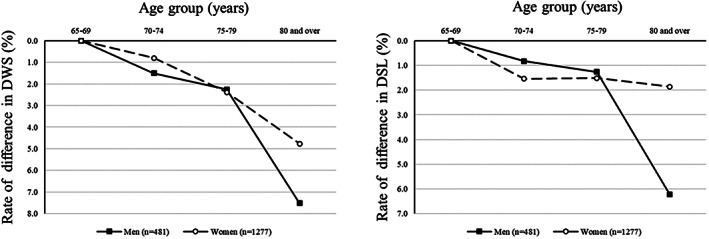
Rate of difference in daily living walking speed (DWS) and daily living step length (DSL) after ages 65–69 years.

As DWS showed significantly greater differences in those aged ≥65 years than in the younger groups, we derived the rate of difference for those aged 65–69 years in DWS and DSL, and compared it with that of a previous study.^29^ Although DWS differed by a maximum of approximately 8% and DSL by a maximum of approximately 7% (Fig. 2), these differences are smaller than those found in the previous study (20–30%). The lower difference rates might have been influenced by the bias toward healthy smartphone users in the older groups.

Conventional walking measurements in laboratories have some important benefits (control over walking environment, duration and distance); however, participants might intentionally alter their walking speed while being observed. Another limitation of laboratory measurement is that participants must visit a measurement site. The present study’s DWS might address problems that arise with measuring WSL. The present reference values and age differences of daily living walking parameters can be compared with the data of daily living walking speed measured by other devices, such as accelerometers. This is useful for establishing a consensus on a DWS definition for assessing older individuals’ health outcomes.

The present study had some limitations, primarily that the participants were all smartphone users, and there might thus be a bias, especially in those aged ≥70 years. As the average walking speed in the laboratory in our other cohort‐based study using smartphone apps was approximately 4–6% slower in non‐participants than in those who actually participated, the reference values in those ages might be somewhat overestimated. Furthermore, as a previous study suggested that walking speed changes seasonally, we used measurements obtained over a 1‐year period.^30^ However, walking speed might have declined in older individuals during this period.

Another limitation is that there are not yet any established DWS parameter cut‐offs for various health problems. For example, although DWS was 1.2 m/s among participants aged ≥85 years in the present study, which is higher than the 1.0 m/s used as a diagnostic criterion for frailty and sarcopenia, there are no formal cut‐off values for frailty and sarcopenia using DWS.^1^, ^2^ Future studies should explore the association between DWS and health outcomes. Additionally, the change in walking speed measured by the application used in the present study might include various factors (neuromuscular function, walking for exercise, walking companion etc.). In the future, it might be fruitful to investigate which gait should be included and how many gait segments are required, according to the purpose of using this measurement.

Walking speed is associated with many health outcomes, and DWS might also be helpful in predicting health outcomes. Furthermore, DWS could have implications for health promotion efforts. Future studies on the association between DWS, health outcomes and applications for health promotion are warranted.

## Disclosure statement

KM is an employee of Taiyo Life Insurance Company, which developed the smartphone application used in this study and provides services using it, but no funds were obtained from the company for this study. The other authors declare no conflict of interest.

## Supporting information


**Figure S1.** Histograms of walking speeds measured in daily life for 12 randomly selected participants.

**Figure S2.** Skewness and kurtosis of the distribution of walking speeds measured in the daily life of all participants.

**Figure S3.** Age‐related differences in daily walking speed (DWS) among men and women.

**Table S1.** Prefectures of the participants
