# Supplementary figures and images for: Reference value on daily living walking parameters among Japanese adults
Source: Geriatr Gerontol Int. 2020 May 6;20(7):664–9. doi: 10.1111/ggi.13931 (PMC7496516; doi:10.1111/ggi.13931)

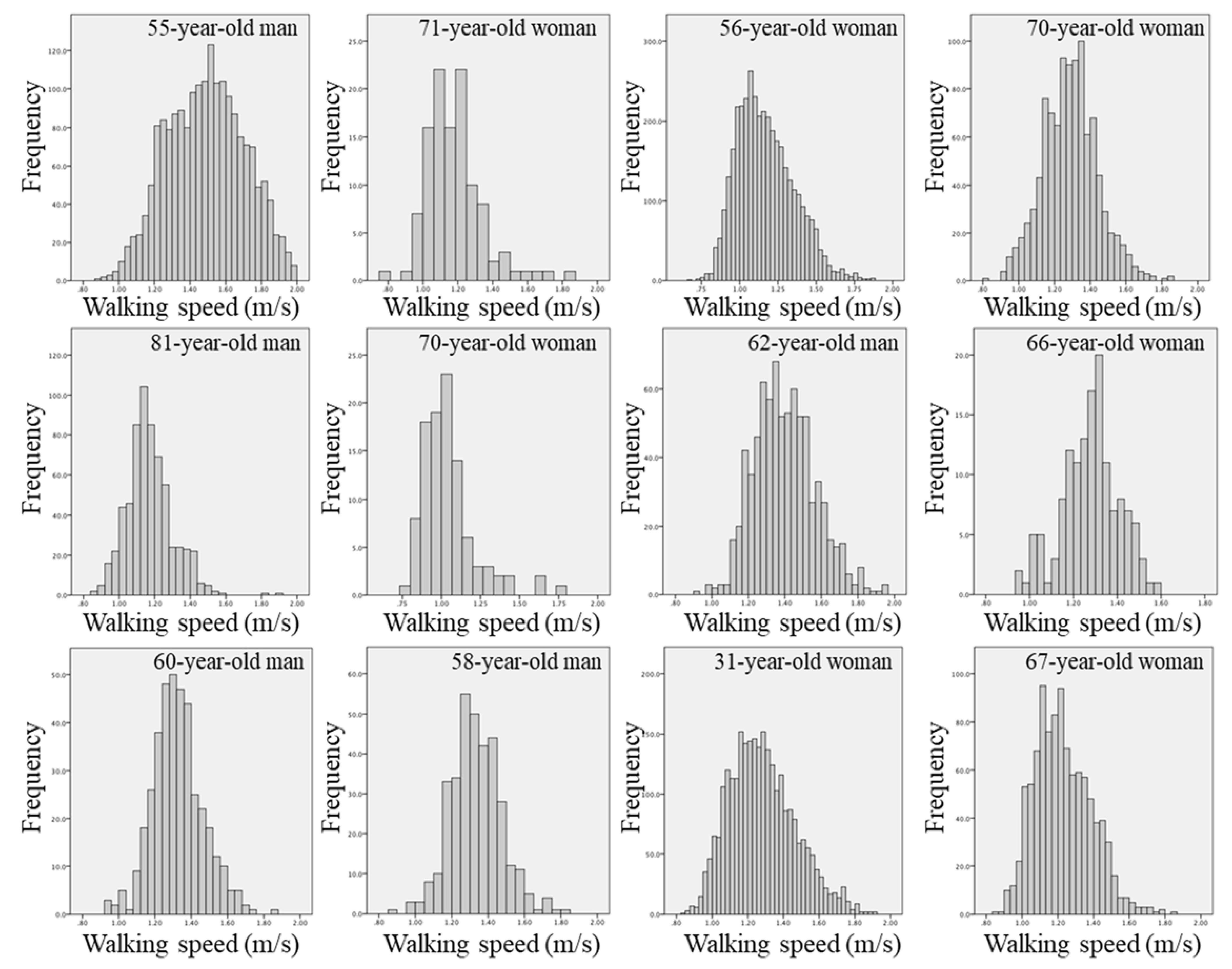

Supplement: Supplementary file 1 — Figure S1. Histograms of walking speeds measured in daily life for 12 randomly selected participants. [file GGI-20-664-s001.jpg]

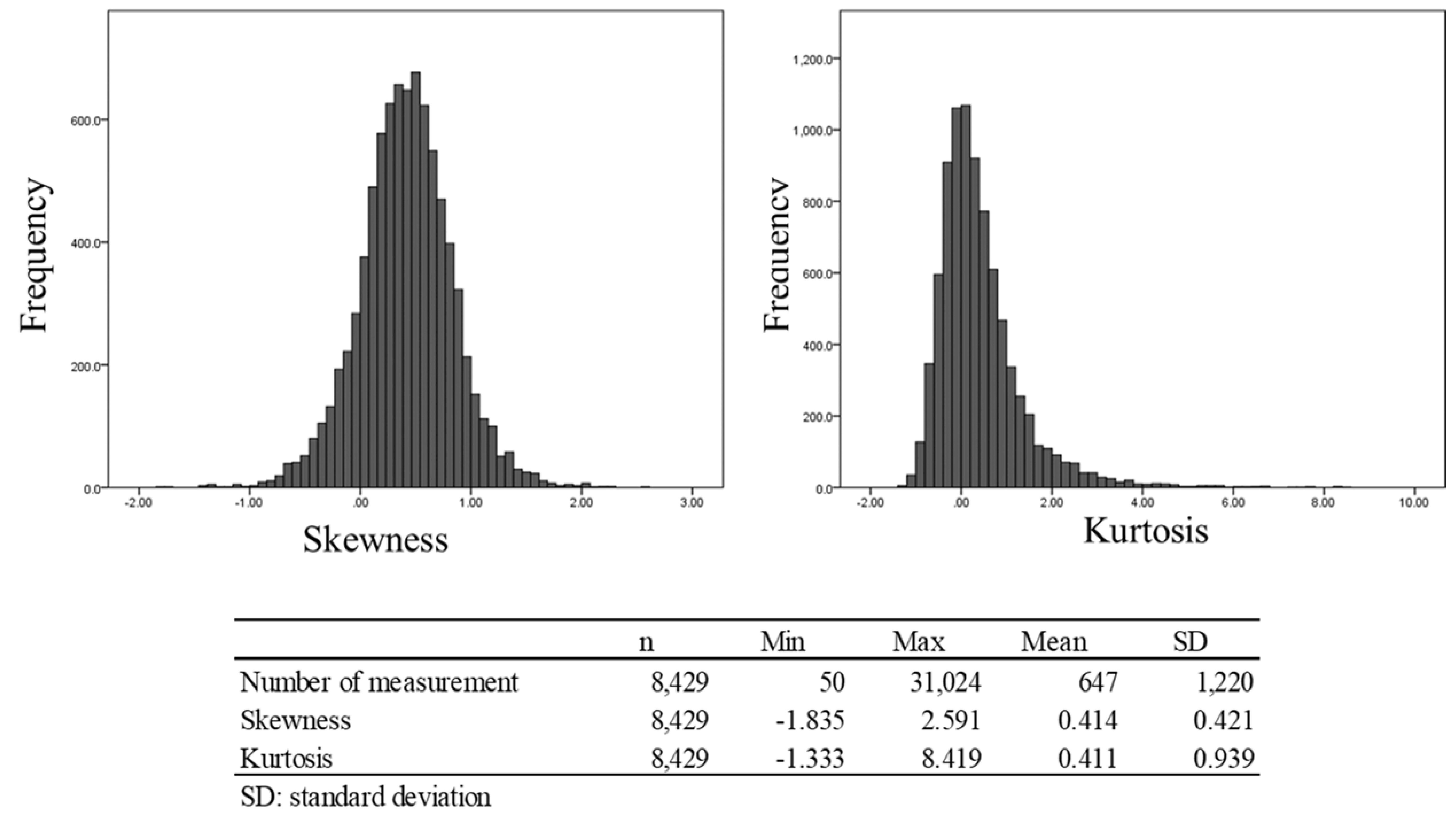

Supplement: Supplementary file 2 — Figure S2. Skewness and kurtosis of the distribution of walking speeds measured in the daily life of all participants. [file GGI-20-664-s003.jpg]

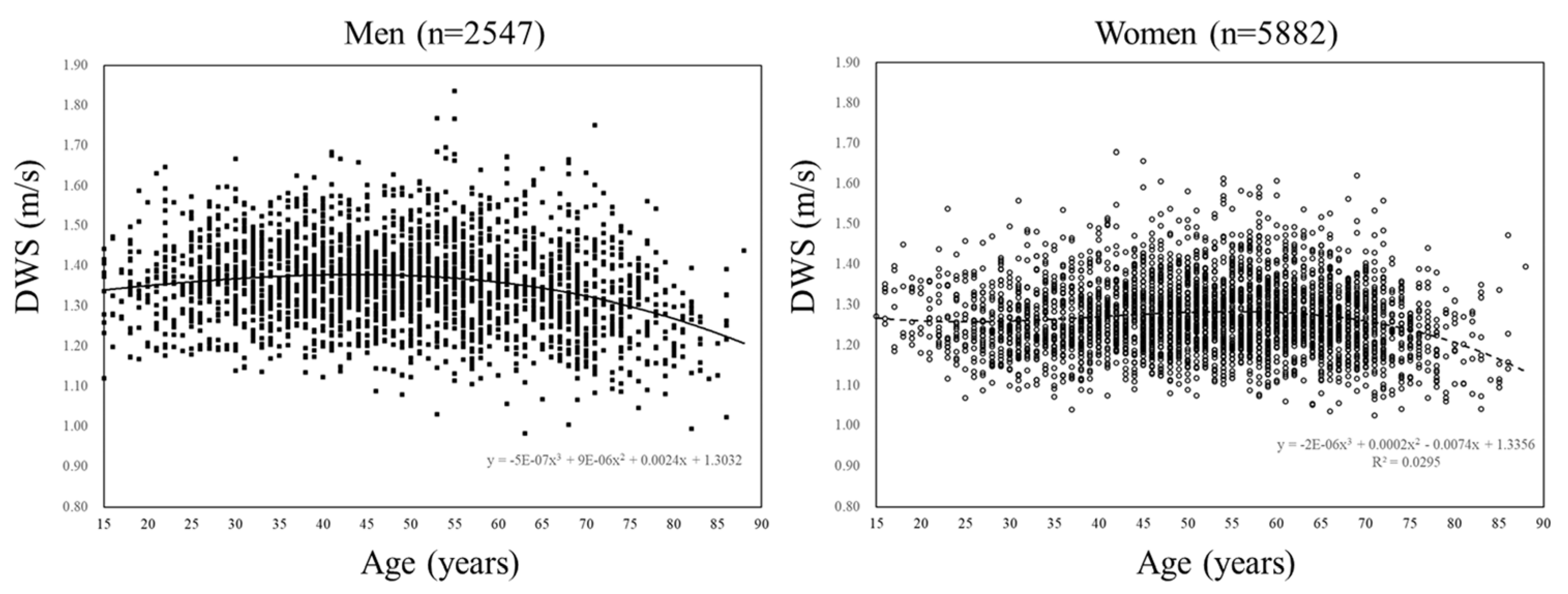

Supplement: Supplementary file 3 — Figure S3. Age‐related differences in daily walking speed (DWS) among men and women. [file GGI-20-664-s004.jpg]
