# Supplementary material for: Reference value on daily living walking parameters among Japanese adults
Source: Geriatr Gerontol Int. 2020 May 6;20(7):664–9. doi: 10.1111/ggi.13931 (PMC7496516; doi:10.1111/ggi.13931)
Supplement: Supplementary file 4 — Table S1. Prefectures of the participants [file GGI-20-664-s002.docx]

**Supporting Table 1.** Prefectures of the participants

| Prefecture | | *n* | % | % of national population |
| --- | --- | --- | --- | --- |
|  | Hokkaido | 328 | 3.9 | 4.3 |
|  | Aomori | 68 | 0.8 | 1.1 |
|  | Iwate | 59 | 0.7 | 1.0 |
|  | Miyagi | 300 | 3.6 | 1.8 |
|  | Akita | 33 | 0.4 | 0.8 |
|  | Yamagata | 50 | 0.6 | 0.9 |
|  | Fukushima | 35 | 0.4 | 1.6 |
|  | Ibaraki | 149 | 1.8 | 2.3 |
|  | Tochigi | 104 | 1.2 | 1.6 |
|  | Gunma | 65 | 0.8 | 1.6 |
|  | Saitama | 517 | 6.1 | 5.6 |
|  | Chiba | 412 | 4.9 | 4.9 |
|  | Tokyo | 711 | 8.4 | 10.3 |
|  | Kanagawa | 794 | 9.4 | 7.1 |
|  | Niigata | 39 | 0.5 | 1.9 |
|  | Toyama | 51 | 0.6 | 0.9 |
|  | Ishikawa | 83 | 1.0 | 0.9 |
|  | Fukui | 13 | 0.2 | 0.6 |
|  | Yamanashi | 5 | 0.1 | 0.7 |
|  | Nagano | 55 | 0.7 | 1.7 |
|  | Gifu | 131 | 1.6 | 1.6 |
|  | Shizuoka | 443 | 5.3 | 2.9 |
|  | Aichi | 486 | 5.8 | 5.8 |
|  | Mie | 64 | 0.8 | 1.4 |
|  | Shiga | 83 | 1.0 | 1.1 |
|  | Kyoto | 250 | 3.0 | 2.1 |
|  | Osaka | 725 | 8.6 | 6.9 |
|  | Hyogo | 287 | 3.4 | 4.4 |
|  | Nara | 39 | 0.5 | 1.1 |
|  | Wakayama | 45 | 0.5 | 0.8 |
|  | Tottori | 1 | 0.0 | 0.5 |
|  | Shimane | 16 | 0.2 | 0.6 |
|  | Okayama | 91 | 1.1 | 1.5 |
|  | Hiroshima | 191 | 2.3 | 2.2 |
|  | Yamaguchi | 134 | 1.6 | 1.1 |
|  | Tokushima | 28 | 0.3 | 0.6 |
|  | Kagawa | 45 | 0.5 | 0.8 |
|  | Ehime | 66 | 0.8 | 1.1 |
|  | Kochi | 33 | 0.4 | 0.6 |
|  | Fukuoka | 424 | 5.0 | 4.0 |
|  | Saga | 42 | 0.5 | 0.7 |
|  | Nagasaki | 156 | 1.8 | 1.1 |
|  | Kumamoto | 112 | 1.3 | 1.4 |
|  | Oita | 41 | 0.5 | 0.9 |
|  | Miyazaki | 49 | 0.6 | 0.9 |
|  | Kagoshima | 70 | 0.8 | 1.3 |
|  | Okinawa | 40 | 0.5 | 1.1 |
|  | Unknown | 466 | 5.5 |  |
|  | Total | 8,429 | 100.0 |  |
